# A case of ulcerated nodules in association with a monoclonal gammopathy

**DOI:** 10.1016/j.jdcr.2024.07.019

**Published:** 2024-08-28

**Authors:** Sheena Bhadresha, Kirsty Cuthill, Jonathan Salisbury, Carolina Fernandez, Danae Trokoudes, Atheer Al Haddabi, Tanya Nandini Basu

**Affiliations:** a Department of Dermatology, Kings College Hospital, London, UK; b Department of Haematology, Kings College Hospital, London, UK; c Department of Histopatholoy, Kings College Hospital, London, UK; d Department of Dermatology, Imperial College Healthcare NHS Foundation Trust, London, UK

**Keywords:** monoclonal gammopathy, necrobiotic xanthogranuloma

## History

**Fig 1:**
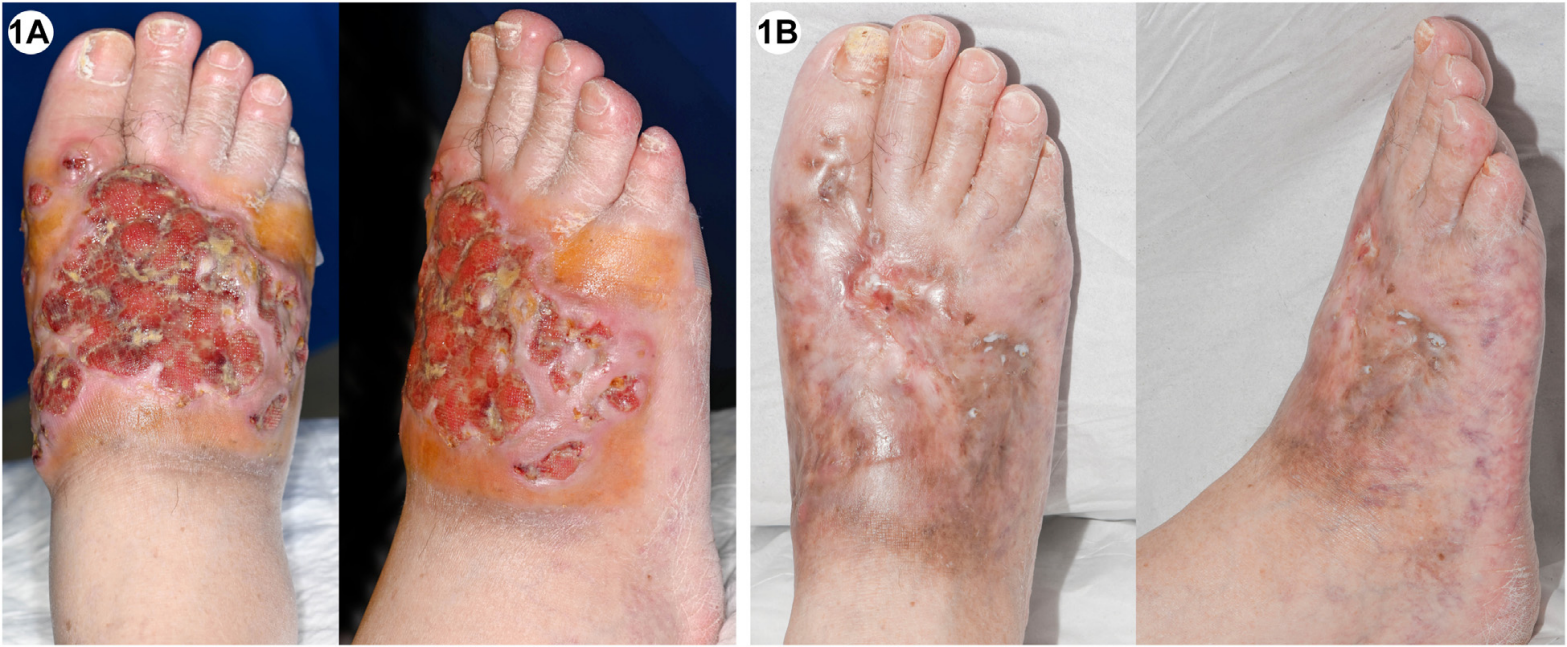

**Fig 2:**
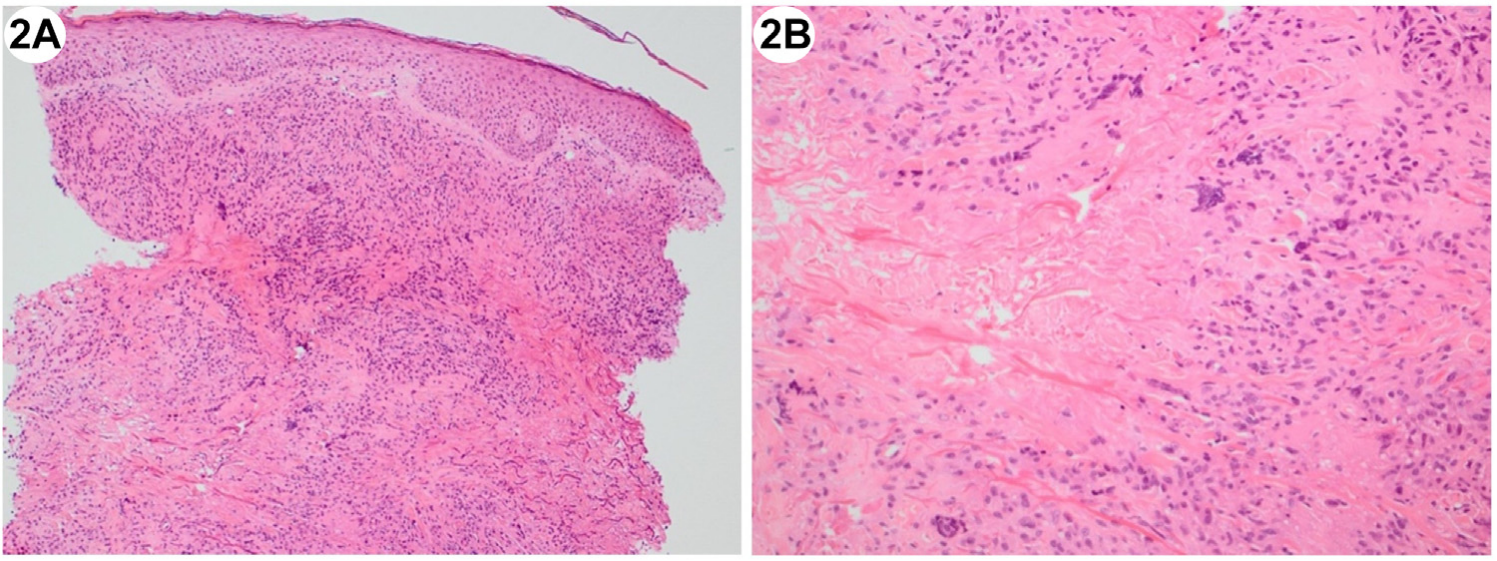

A 75-year-old man with a monoclonal gammopathy of undetermined significance presented with a 10-year history of painful ulcerated nodules on the dorsum of the right foot (Fig 1, *A*). Skin biopsy of a similar plaque on the left arm showed necrobiosis with multinucleated giant cells (Fig 2, *A* and *B*). Blood tests confirmed an IgG kappa paraproteinemia of 20 g/L and normal serum lipid profile. Bone marrow biopsy revealed transformation to smoldering myeloma. Treatment with eight cycles of Bortezomib, Cyclophosphamide, and Dexamethasone resulted in resolution of skin signs (Fig 1, *B*), pain, and a reduction in the paraproteinemia to <1 g/L.

**Question 1: What is the most likely underlying diagnosis?**

A. Necrobiotic xanthogranuloma
B. Necrobiosis lipoidica
C. Granuloma annulare
D. Juvenile xanthogranuloma
E. Foreign-body granuloma

**Answers:**

A. Necrobiotic xanthogranuloma – Correct. Necrobiotic xanthogranuloma is a non-Langerhans histiocytosis. Cutaneous plaques and nodules commonly affect the torso, periorbital, and acral sites. Eighty-four percent of patients with necrobiotic xanthogranuloma have a monoclonal gammopathy of undetermined significance.^1^
B. Necrobiosis lipoidica – Incorrect. This is associated with diabetes and not associated with a serum paraproteinemia. Necrobiosis lipoidica classically affects the pretibial area.
C. Granuloma annulare – Incorrect. Typically affects dorsa of the hands, feet, and ankles. Palisading granulomas and presence of mucin can be seen on histological analysis.
D. Juvenile xanthogranuloma – Incorrect. Commonly affects infants and young children.
E. Foreign-body granuloma – Incorrect. There was no history of trauma or entry of exogenous material into the skin.

**Question 2: Necrobiotic xanthogranuloma is most commonly associated with which condition?**

A. Hyperthyroid
B. Inflammatory bowel disease
C. Myeloid blood dyscrasias
D. Paraproteinemia
E. Rheumatoid arthritis

**Answers:**

A. Hyperthyroid – Incorrect. Uncontrolled hyperthyrodism may be associated with the development of pretibial myxedema.
B. Inflammatory bowel disease – Incorrect. Inflammatory bowel disease is associated with pyoderma gangrenosum.
C. Myeloid blood dyscrasias – Incorrect. This is associated with pyoderma gangrenosum.
D. Paraproteinemia – Correct. Necrobiotic xanthogranuloma is associated with plasma cell dyscrasias, particularly Ig-G kappa proteinaemias.^2^
E. Rheumatoid arthritis – Incorrect. Rheumatoid arthritis may be associated with rheumatoid nodules.

**Question 3: Which statement is true regarding the clinical features of necrobiotic xanthogranuloma?**

A. Necrobiotic xanthogranuloma can present with periorbital plaques in the skin but does not result in ocular manifestations.
B. Necrobiotic xanthogranuloma often presents as a solitary nodule or plaque in the skin.
C. Necrobiotic xanthogranuloma only affects the skin.
D. Plaques of necrobiotic xanthogranuloma do not exhibit telangiectasia.
E. Scarring and ulceration of necrobiotic xanthogranulomatous plaques occur in nearly half of patients.

**Answers:**

A. Necrobiotic xanthogranuloma can present with periorbital plaques in the skin but does not result in ocular manifestations – Incorrect. Ocular manifestations can occur in about 50% of patients. They include burning, itching, blurred vision, diplopia, and acute transient loss.^3^
B. Necrobiotic xanthogranuloma often presents as a solitary nodule or plaque in the skin – Incorrect. Cutaneous plaques and nodules commonly affect the torso, periorbital, and acral sites.
C. Necrobiotic xanthogranuloma only affects the skin – Incorrect. It may involve extracutaneous sites, including lung, myocardium, larynx, ovary, and intestine.^4^
D. Plaques of necrobiotic xanthogranuloma do not exhibit telangiectasia – Incorrect. Lesions often show superficial telangiectasias.^5^
E. Scarring and ulceration of necrobiotic xanthogranulomatous plaques occur in nearly half of patients – Correct. Debilitating ulceration can be painful and lowers the threshold for treatment. Plasma-cell-directed therapies that are well tolerated and highly effective at reducing paraprotein levels can be extremely effective in treating monoclonal gammopathies of cutaneous significance, even when hematological criteria for treatment are not met.

## Conflicts of interest

None disclosed.
